# The impact of vanishing white matter on unaffected family members

**DOI:** 10.1186/s13023-025-03987-8

**Published:** 2025-08-26

**Authors:** Romy J. van Voorst, Daphne H. Schoenmakers, Irene van Beelen, Francesco Gavazzi, Alexandra Chapleau, Adeline Vanderver, Geneviève Bernard, Ingeborg Krägeloh-Mann, Marjo S. van der Knaap

**Affiliations:** 1 Department of Pediatric Neurology, Emma Children’s Hospital, Amsterdam University Medical Centre, Amsterdam, The Netherlands; 2 Amsterdam Leukodystrophy Center, Amsterdam Neuroscience, Cellular & Molecular Mechanisms, Amsterdam, The Netherlands; 3 Department of Endocrinology and Metabolism, Platform “Medicine for Society”, Amsterdam UMC Location University of Amsterdam, Amsterdam, The Netherlands; 4 Division of Child Neurology, Department of Pediatrics, Children’s Hospital of Philadelphia, Philadelphia, PA USA; 5 Departments of Neurology and Neurosurgery, Pediatrics and Human Genetics, McGill University, Montreal, Canada; 6 Child Health and Human Development Program, Research Institute of the McGill University Health Centre, Montreal, QC Canada; 7 Department of Neurology, Perelman School of Medicine, University of Pennsylvania, Philadelphia, PA USA; 8 Department of Specialized Medicine, Division of Medical Genetics, McGill University Health Centre, Montreal, Canada; 9 Child Health and Human Development Program, McGill University Health Centre Research Institute, Montreal, Canada; 10 Department of Developmental and Child Neurology, Social Pediatrics, University Children’s Hospital Tübingen, Tübingen, Germany; 11 Department of Integrative Neurophysiology, Center for Neurogenomics and Cognitive Research, Vrije Universiteit Amsterdam, Amsterdam, The Netherlands

**Keywords:** Leukodystrophy, Vanishing white matter, VWM, Ultra-rare diseases, Impact of disease, Quality of life, Family members

## Abstract

**Background:**

Vanishing White matter (VWM) is one of the more prevalent leukodystrophies, caused by biallelic pathogenic variants in any of the *EIF2B1–5* genes. It is characterized by chronic progressive neurological deterioration and additional stress-provoked episodes of rapid decline, leading to severe neurological impairment and early death. The impact of VWM on unaffected family members has not been investigated.

**Methods:**

This international cross-sectional study enrolled parents, partners, and unaffected siblings. We used online administration of (1) health-related quality of life questionnaires (quantitative, comprising the EuroQol–5-Dimensions [EQ5-D]–5-Levels questionnaire [EQ-5D-5L], EuroQol–5-Dimensions–Youth–3-Levels questionnaire [EQ-5D-Y-3L], Pediatric Quality of Life Family Impact Module [PedsQL™-FIM], PedsQL™ Child–Adult Self Report [PedsQL™-SC]); (2) VWM-specific customized questionnaires (quantitative, comprising the impact of VWM inventory questionnaires for parents, partners and siblings); and (3) in-depth semi-structured interview (qualitative).

**Results:**

A total of 100 family members were included: 52 mothers, 29 fathers, 13 unaffected siblings, and 6 partners. Mothers and partners scored significantly poorer on the EQ5D-5L than the reference norms. Fathers and mothers scored significantly poorer on the PedsQL™-FIM than the reference norms. Siblings scored similar to the reference norms on the EQ5D-5L and all domains of the PedsQL™-SC, with the lowest score on the emotional domain. Qualitative interviews revealed three main drivers of the impact of VWM: (1) lack of knowledge and empathy of healthcare professionals, (2) unpredictable disease course, and (3) caregiver responsibilities. Mothers reported substantial impacts on their emotional well-being and dissatisfaction with their professional development. Fathers commonly reported financial concerns and heightened family responsibility. Partners mentioned emotional exhaustion and difficulty in managing family responsibilities. Siblings frequently reported internal struggles, finding it challenging to express their feelings.

**Conclusions:**

Mothers and partners indicate a significant and consistent reduction in their quality of life on standardized questionnaires. Qualitative interviews revealed in-depth details of VWM’s impact on all family members. Improved healthcare communication, symptom management resources, and support networks are essential for alleviating VWM’s impact on families. This study emphasizes the importance of tailored approaches to supporting family members of VWM patients and enhancing their quality of life.

**Supplementary Information:**

The online version contains supplementary material available at 10.1186/s13023-025-03987-8.

## Introduction

Vanishing White Matter (VWM) is one of the more prevalent leukodystrophies, characterized by chronic neurological deterioration [1]. It is caused by biallelic pathogenic variants in any of the *EIF2B1–5* genes [2]. These genes encode the subunits of eukaryotic translation initiation factor 2B (eIF2B), a factor that is conditional for translation of mRNAs into proteins and regulates the integrated stress response (ISR) [3]. The eIF2B defect causes dysregulation of the ISR [4]. As a result, patients are highly susceptible to stressors, such as febrile infections or minor head trauma, often resulting in rapid neurological decline, lowered consciousness, and sometimes death [5, 6]. Recovery of such episodes is frequently incomplete, leading to permanent loss of neurological functions.

VWM can present at all ages, ranging from antenatal period to senescence [1]. Disease progression is highly variable between patients [5]. Age of onset is the best predictor of disease progression; patients with early onset have faster and more severe disease progression than patients with later onset [5]. In the classic phenotype, the disease manifests between 2 and 6 years of age. Often, a stress-provoked episode of rapid decline marks the disease onset [5]. Symptoms of the classic phenotype are dominated by cerebellar ataxia, and cognition is relatively spared. Onset in adulthood is associated with slower decline, and stress-provoked episodic deterioration is less prominent [5]. These patients typically present with cognitive regression or psychiatric symptoms and often have late and mild motor involvement.

No disease-modifying therapies are currently available, and treatment is limited to symptomatic management. For most patients, preventive strategies, such as minimizing exposure to infections, are implemented by families to minimize the risk of episodes of rapid deterioration. Several potential therapeutic targets within the ISR have been identified. Two compounds, Guanabenz (EudraCT 2027-001438-25, CTIS 2023-503320-89-00) and ABBV-CLS-7262 (NCT05757141), which activate eIF2B either indirectly or directly, respectively, have shown beneficial effects in a representative VWM mouse model and are currently being tested in clinical trials [7, 8].

Patients with VWM require various types of supportive care throughout their lives. Previous studies on severe, life-limiting illnesses have shown that such conditions affect not only patients but also their families [9, 10]. Caregivers often face considerable challenges, a phenomenon observed across different rare disease contexts in multi-country studies [10, 11]. The impact of VWM on the family system has not been investigated. A comprehensive assessment of the impact of VWM on families is required for evaluating new therapies to ensure they sufficiently address the needs of patients and their families.

## Methods

### Study design

This international cross-sectional study enrolled parents, partners, and unaffected siblings 12 years of age or older. Proficiency in either Dutch or English was required. Relatives or partners of deceased VWM patients were excluded. If a family had multiple patients, relatives were asked to respond regarding all. Participants were required to live in the same household during part of the disease course. Live-in partners of parents of VWM patients were invited if they were also care providers. We used a three-step approach to acquire both quantitative and qualitative information: (1) online administration of health-related quality of life questionnaires (quantitative, comprising the EuroQol–5-Dimensions [EQ5-D]–5-Levels questionnaire [EQ-5D-5L, version 1.2] or EuroQol–5-Dimensions–Youth–3-Levels questionnaire [EQ-5D-Y-3L, version 2.2], and Pediatric Quality of Life Family Impact Module [PedsQL™-FIM, version 2.0] or PedsQL™ Child–Adult Self Report [PedsQL™-SC, version 4.0]); (2) online administration of VWM-specific customized questionnaire (quantitative and qualitative, comprising the impact of VWM inventory questionnaires for parents, partners and unaffected siblings); and (3) in-depth semi-structured interview (qualitative). All participants were instructed to complete the questionnaires separately. Data collection was conducted over a 12-month-period.

### Participant recruitment and consent

Participants were recruited through an international registry for VWM, which uses online questionnaires to track disease progression and clinical characteristics of VWM patients, such as age, age at onset, disease duration, symptoms, and number of affected and unaffected siblings. Informed consent from parents and partners was secured through this registry. Extra consent was required for unaffected siblings. For siblings under the age of 16, both the sibling and the parents had to sign the informed consent, while for siblings from the age of 16, the sibling’s consent alone sufficed.

### Questionnaires

#### Self-reported EQ-5D-5L/Y-3L

The EQ-5D assesses the quality of life of family members across five health dimensions: Mobility, Self-Care, Usual Activities, Pain/Discomfort, and Anxiety/Depression. The dimensions of the EQ5D-5L are scored into five levels (no, slight, moderate, or severe problems, or unable). The dimensions of the EQ-5D-Y-3L are scored into three levels, (no, some, or a lot of problems). The level of problems reported on all EQ-5D dimensions together determines a unique health state. Health states are converted into a weighted health state index by applying scores from the EQ-5D preference weight, obtained from general population samples. EuroQol Research Foundation recommends using the United States of America (U.S.A.) EQ-5D-5L value set from the study of Pickard and colleagues [12] for calculating index values for countries, for which no values set has been published. Given the rarity of VWM and the international recruitment of this study, we used the value set of Pickard and colleagues as reference norms. The weighted health state index is converted into EQ-5D Utility Index scores, ranging from 0 (indicating death) to 1 (representing perfect health). Additionally, the EQ-5D includes a visual analog scale (VAS), asking family members to rate their overall current health on a scale of 0 to 100, where 0 signifies the worst imaginable health and 100 the best imaginable health.

#### PedsQL™-FIM and PedsQL™-SC

The impact of VWM on family functioning was evaluated using the PedsQL™-FIM. Parents were instructed to fill out the questionnaire based on their experiences from the past month as well as from the past 7 days. The questionnaire consists of 36 items across the following domains: Physical Functioning (6 items), Emotional Functioning (5 items), Social Functioning (4 items), Cognitive Functioning (5 items), Communication (3 items), Worry (5 items), Daily Activities (3 items) and Family Relationships (5 items). Items are reversed-scored and linearly transformed on a scale from 0 to 100 (0 = 100; 1 = 75; 2 = 50; 3 = 25; 4 = 0). Scale scores are only calculated if at least 50% of the items in the respective scale have been answered. The parent’s Health-Related Quality of Life Summary Score (HRQoL-SS) is calculated by the sum of the items Physical, Emotional, Social, and Cognitive Functioning domains (20 items) divided by the number of items answered. The Family Functioning Summary Score (FFSS) (8 items) is calculated by the sum of the items from the Daily Activities and Family Relationships domains (8 items in total), divided by the number of items answered. The Total Scale Score is calculated by the sum of all 36 items divided by the number of items answered. We compared the results of the PedsQL™-FIM to those of a community-based sample of families in the U.S.A., and within this sample, specifically families with children without “chronic medical conditions” [13].

The PedsQL™ is not suitable for partners of patients. Siblings were asked to complete the PedsQL™-SC based on their age group (13- < 18 years, 18- < 25 years, or ≥ 25 years) with a recall period of the past month and the past 7 days. Domains of this self-report include: Physical Functioning (8 items), Emotional Functioning (5 items), Social Functioning (5 items), and School/Work Functioning (5 items). The Psychosocial Health Summary Score (15 items) is computed as the sum of the items over the number of items answered in the Emotional, Social, and School/Work Functioning domains. The Physical Health Summary Score is the same as the Physical Functioning domain. The Total Scale Score is computed as the sum of all 23 items divided by the number of items answered on all domains. We compared the results of the PedsQL™-SC 13- < 18 years, 18- < 25 years, and ≥ 25 years to those of healthy individuals of the same age groups in The Netherlands [14, 15]. Comprehensive U.S.A. reference norms exist for ages 13– < 18, but are limited for ages ≥ 18 years, relying mainly on student samples. Dutch PedsQL™-SC reference norms cover ages 18– < 25 years and ≥ 25 years, with the scale validated for use up to the ages of 30 years. We also used the Dutch reference norms for the 13– < 18 age group for consistency.

#### Impact questionnaire and semi-structured interviews

We adapted the questionnaire used for metachromatic leukodystrophy (MLD) by Ammann‑Schnell and colleagues [9] to develop the impact of VWM inventory questionnaires for parents, partners, and unaffected siblings (Supplementary Material 1–3). The questions refer to the impact of the diagnosis, communication, and level of disease-specific knowledge of healthcare professionals, family and caregiving, professional development, social support, and participant’s satisfaction with their understanding of the disease. The questionnaires include a combination of quantitative and qualitative open-ended questions. A 5-point Likert scale is used to quantify opinions and attitudes regarding specific situations or topics metrically. The open-ended questions allow participants to elaborate on these aspects and discuss issues not covered by the standardized questions.

Semi-structured interviews, following the order of the questions on the impact of the VWM inventory questionnaire, were recorded using Microsoft Teams (v19.1.8), and the recordings were subsequently transcribed. The software tool MAXQDA (MAXQDA Plus 2022, release 22.1.1) was used to analyze the interviews qualitatively. Two researchers (RJvV and DHS) separately analyzed two interviews and compared codes for training purposes; codes were discussed for discrepancies or uncertainties, and consensus was reached. After that, interviews were coded by one of the researchers (either RJvV or DHS), who discussed cases of doubt and reached agreement. A combined deductive and inductive coding approach was used to code all interview segments related to the impact of VWM. Deductive codes were based on the questions of the impact of the VWM inventory questionnaire, while inductive codes emerged from the coding process. Transcripts were coded until no new codes emerged in four sequential interviews, and saturation was reached. Similar codes were grouped to identify overarching themes. The results were structured to first present the main themes derived from the collective insights of the family members during the semi-structured interviews. Subsequently, quantitative findings of the impact inventory were grouped by specific family groups: parents, partners, and siblings. These quantitative results were enriched with qualitative data and illustrative quotes from the interviews.

### Statistical analysis

Mean and standard deviation (SD) were calculated for continuous data and proportions were reported as n (%). Median and interquartile range (IQR) were calculated for smaller sample sizes and non-normally distributed data. Data analysis and visualization were performed using R Studio for Windows Version 24.0 (IBM Corp., Armonk, NY, U.S.A.). A *p*-value of < 0.05 was considered statistically significant. The demographic EQ5D-5L dimension scores of the different family members were compared to the reference norms [12] using a Fisher’s exact test. The one sample Wilcoxon-signed-rank test was used to compare the family members’ VAS and Utility Index scores with the reference norms. A sensitivity analysis was performed comparing the value sets of the U.S.A. [12] and The Netherlands [16] to assess the robustness of the Utility Index score. Due to the absence of established EQ5D-Y-3L reference norms, data interpretation was not feasible.

The PedsQL™-FIM domain and summary community-based reference norms, as well as the norms based on families with children without a chronic medical condition, are available for a recall period of the past month, so only scores over the past month could be compared to these reference norms [13]. A one-sample t-test compared the parents’ PedsQL™-FIM summary scores over the past month to the reference norms based on families with children without a chronic medical condition. A paired samples t-test compared parents’ summary scores between the past month and the past 7 days. PedsQL™-SC domain and summary scores for ages 13– < 18, 18– < 25, and ≥ 25 years were analyzed and compared to the reference norms in a comparable manner.

To investigate the impact of communication during diagnosis by healthcare professionals, an independent t-test was used to compare the mean scores of the PedsQL™-FIM over the past month between the satisfied (Likert scale 3–4) and less satisfied (Likert scale 0–1) sample. Additionally, median differences between parents in professional satisfaction and level of financial worry (items from the impact of the VWM questionnaire) were investigated using a Wilcoxon sum rank test.

## Results

### Demographics participants

Of the 147 family members contacted through the VWM registry, 100 participants from 63 families completed the online survey, comprising 81 parents (52 mothers, 29 fathers), 13 unaffected siblings, and 6 partners. Participants represented 21 countries: U.S.A. (n = 26), Netherlands (n = 22), Brazil (n = 10), Spain (n = 5), Belgium (n = 4), Germany (n = 4), United Kingdom (n = 4), Australia (n = 3), Chile (n = 2), Czech (n = 2), France (n = 2), Ireland (n = 2), Italy (n = 2), Portugal (n = 2), Russia (n = 2), Turkey (n = 2), Canada (n = 1), Norway (n = 2), Qatar (n = 1), Romania (n = 1), and Saint-Lucia (n = 1). Parents had a mean age of 45.3 years (SD = 11.0), siblings of 25.4 (SD = 12.7), and partners of 52.4 (SD = 11.2). Sex distribution was equal among partners and siblings, while more females (64%, 52/81) participated in the parent group (Table 1).

**Table 1:** Demographics

Variable	Parents	Unaffected siblings	Partners
Participant (n/N)	81/100	13/100	6/100
Sex (male%)	36% (29/81)	54% (7/13)	50% (6/6)
Continent (%)			
Europe	52% (42/81)	69% (9/13)	50% (3/6)
North-America	28% (23/81)	23% (3/13)	17% (1/6)
South-America	15% (10/81)	8% (1/13)	17% (1/6)
Other	7% (6/81)	0% (0/13)	17% (1/6)
Age of the unaffected family member			
Mean (± SD)	45.3 (11.0)	25.4 (12.7)	52.4 (11.2)
Median (IQR)	43.6 (38.0–50.3)	23.6 (14.9–29.1)	55.6 (44.6–61.5)
Multiple affected individuals (yes%)	17% (14/81)	31% (4/13)	NA
Multiple unaffected individuals (yes%)	73% (59/81)	69% (9/13)	NA
VWM patients (n/N)	67/74	15/74	6/74
Age			
Mean (± SD)	14.0 (± 9.9)	25.2 (± 16.6)	51.9 (± 13.2)
Median (IQR)	12.0 (6.7– 17.3)	20.0 (13.5–32.6)	54.3 (43.0–60.8)
Adult patients (%)	22% (15/67)	53% (8/15)	100% (6/6)
Sex (male%)	57% (38/67)	47% (7/15)	50% (3/6)
Disease duration			
Mean (± SD)	9.7 (± 8.8)	15.7 (± 10.0)	11.3 (± 7.9)
Median (IQR)	7.4 (2.7–14.1)	14.0 (10.9–18.2)	12.0 (4.5–15.3)
Age of disease onset			
Mean (± SD)	4.3 (± 3.5)	9.5 (± 9.4)	40.6 (± 18.8)
Median (IQR)	3.0 (2.0–5.0)	5.5 (4.0–13.5)	46.5 (25.0- 49.3)
Episodes of rapid decline (yes%)	70% (47/67)	80% (12/15)	100% (6/6)
Loss of walking without support (yes%)	69% (46/67)	73% (11/15)	50% (3/6)
Loss of walking with support (yes%)	48% (32/67)	40% (6/15)	33% (2/6)
Tube feeding (yes%)	13% (9/67)	0% (0/15)	0% (0/6)
Cognitive decline (yes%)	42% (28/67)	67% (10/15)	83% (5/6)
Seizures (yes%)	34% (23/67)	20% (3/15)	33% (2/6)
Behavioral problems (yes%)	46% (31/67)	44% (7/15)	50% (3/6)

N, number; N, total number; NA, Not Applicable; SD, standard deviation; IQR, interquartile range

### Demographics VWM patients

Details are provided in Table 1. The mean age of the patients was 14.0 years (SD = 9.9) in the parent group, 25.2 years (SD = 16.6) in the sibling group, and 51.9 years (SD = 13.2) in the partner group. In the parent group, the mean age of disease onset of the patients was 4.3 years (SD = 3.5), in the sibling group it was 9.5 years (SD = 9.4), and in the partner group the mean age of onset of patients was 40.6 years (SD = 18.8). In the parent group, the mean disease duration of patients at the time of the interview was 9.7 years (SD = 8.8), in the sibling group it was 15.7 years (SD = 10.0), and in the partner group it was 11.3 years (SD = 7.9). The sex distribution of the patients was equal across all groups. Nearly all patients with VWM (88%, 65/74) had experienced episodes of rapid decline. In the parent group, a substantial proportion of patients was immobile (48%, 32/67), with some requiring tube feeding (13%, 9/67). Patients in the partner group were commonly reported to have cognitive impairments (83%, 5/6). The prevalence of seizures and behavioral issues was similar across the groups.

### Mothers and partners report poor EQ-5D health outcomes

**Fig. 1:**
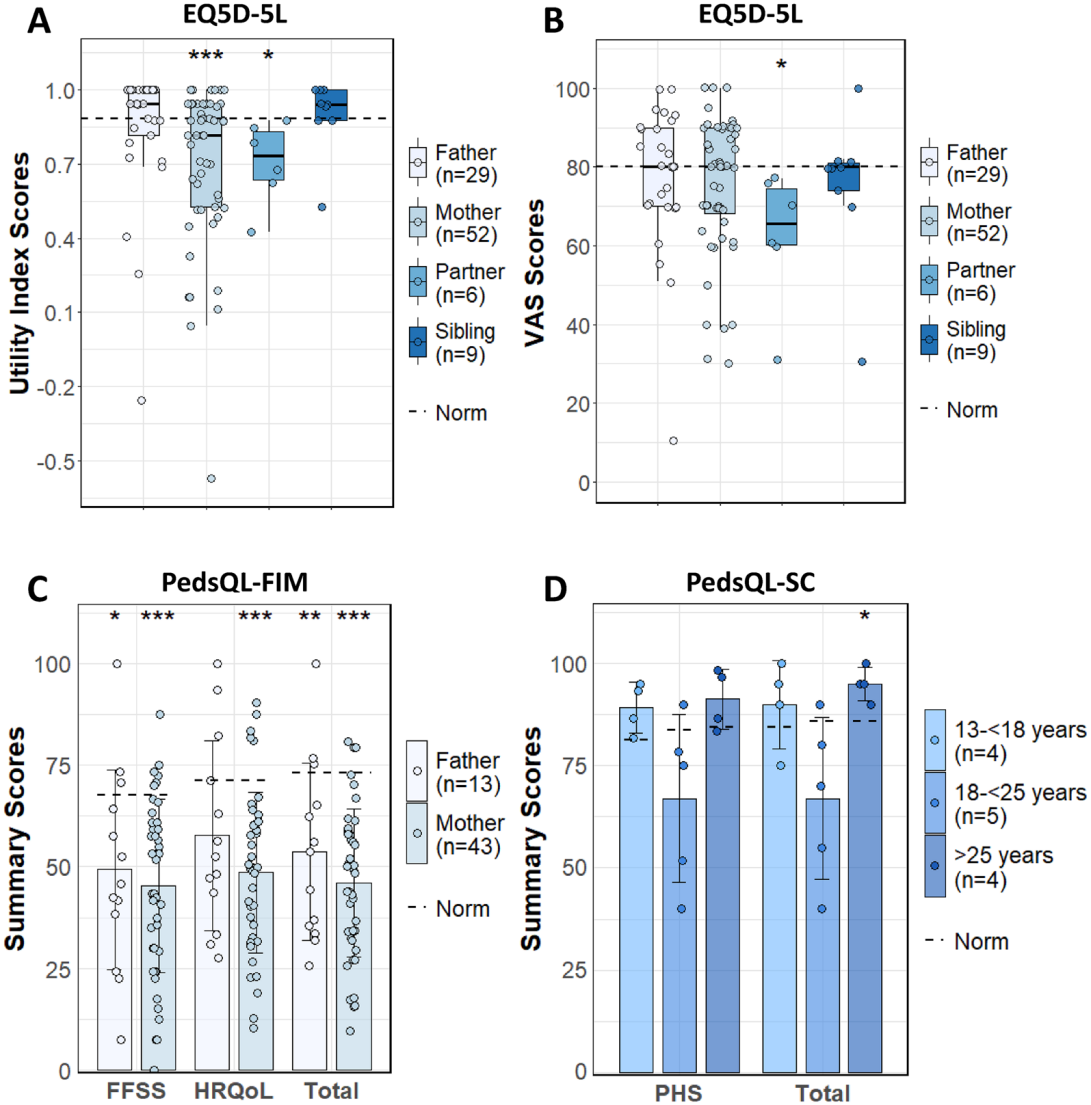
Quality of life scores of unaffected family members compared to norms over the past month. *Note*. **A** EQ5D-5L Utility Index scores by unaffected family members. **B** EQ5D-5L VAS scores by unaffected family members. **C** PedsQL™-FIM scores by parents. FFSS = Family Functioning Summary Score; HRQoL = Health-Related Quality of Life Summary Score; Total = Total Scale Score. **D** PedsQL™-SC scores reported by siblings, categorized by age. PHS = Psychosocial Health Score; Total = Total Scale Score. The dashed line represents the reference norms for these scores. Statistical significance is indicated as follows: *p < 0.05, **p < 0.01, ***p < 0.001

Details are provided in Table 2, Fig. 1 and Supplementary Table 1. Mothers and partners had significantly poorer Utility Index scores than the U.S.A. reference norms (*p* < 0.001 and *p* < 0.05, respectively); the scores of siblings and fathers did not significantly differ from those of the reference norms. Sensitivity analysis revealed minor differences between The Netherlands and The U.S.A. Utility Index scores (Supplementary Table 2), indicate the robustness of this score. Partners scored significantly poorer on the VAS than the reference norms (*p* < 0.05); there were no significant differences on the VAS between the reference norms and the other family members. Mothers (*p* < 0.005) and partners (*p* < 0.001) scored poorer on the Anxiety and Depression domain of the dimension-specific scores than the reference norms. Conversely, fathers (*p* < 0.05) and siblings (*p* < 0.05) scored significantly better than the reference norms in the Pain and Discomfort domain.

**Table 2:** EQ5D Utility Index, VAS, and dimension scores of unaffected family members versus norms

EQ5D Scores	Fathers (n = 29)	Mothers (n = 52)	Partners (n = 6)	Unaffected Siblings (≥ 18 years) (n = 9)
Utility Index^1^	0.94 (0.82–1.0)	0.82 (0.53–0.94)	0.73 (0.64–0.83)	0.94 (0.88–1.0)
Reference Norm^3^	0.88	0.88	0.88	0.88
P-Value	> 0.05	**< 0.001**	**< 0.05**	> 0.05
VAS^1^	80.0 (70.0–90.0)	80.0 (68.2–90.0)	65.5 (60.2–74.5)	80.0 (74.0–81.0)
Reference Norm	80.0	80.0	80.0	80.0
P-Value	> 0.05	> 0.05	**< 0.05**	> 0.05
Mobility^2^	17.4%	23.1%	33.3%	11.1%
Reference Norm	29.4%	29.4%	29.4%	29.4%
P-Value	> 0.05	> 0.05	> 0.05	> 0.05
Self-Care^2^	10.7%	17.3%	16.7%	11.1%
Reference Norm	13.9%	13.0%	13.0%	13.0%
P-Value	> 0.05	> 0.05	> 0.05	> 0.05
Usual Activities^2^	24.2%	38.5%	50.0%	33.3%
Reference Norm	31.2%	31.2%	31.2%	31.2%
P-Value	> 0.05	> 0.05	> 0.05	> 0.05
Pain/Discomfort^2^	38.0%	55.8%	66.7%	22.2%
Reference Norm	62.9%	62.9%	62.9%	62.9%
P-Value	**< 0.05**	> 0.05	> 0.05	**< 0.05**
Depression/Anxiety^2^	44.9%	73.1%	100.0%	44.4%
Reference Norm	51.1%	51.1%	51.1%	51.1%
P-Value	> 0.05	**< 0.005**	** < 0.001**	> 0.05

^1^Data presented as Median (IQR). ^2^Data presented as a percentage reporting any problems on the dimension. ^3^Reference norms are based on the U.S.A. EQ5D-5L scores. Significant differences from the reference norms are marked in bold

VAS, visual analog scale

### Parents reported poor summary scores on the PedsQL™-FIM

Details are provided in Table 3 and Fig. 1. Parents scored poorer than the reference norms on all domains over the past month and 7 days, with the poorest scores in the Daily Activities domain over the past month and in the Worry domain over the past 7 days. Fathers scored higher than mothers on all domains over the past one month and past 7 days, except for poorer scores in the Family Functioning domain over the past month. Compared to the reference norms, fathers and mothers scored significantly poorer on the FFSS (*p* < 0.05 and *p* < 0.001, respectively) and the Total Scale Score (*p* < 0.01 and *p* < 0.001, respectively) over the past month. Mothers scored significantly poorer on the HRQoL-SS (*p* < 0.001) than the reference norms over the past month, while fathers did not score significantly different from the reference norms.

**Table 3:** PedsQL™-FIM domain and summary scores parents versus norms: past month and past 7 days

PedsQL™-FIM	Domain Scores	Fathers (n = 13)	Reference Norms^1^	Mothers (n = 43/n = 42) ^6^	Reference Norms^1^
Past one month / past 7 days	Physical Functioning	57.7 (25.2) / 65.1 (29.9)	64.9 (17.4) /–	47.1 (20.5) / 51.2 (23.7)	64.9 (17.4) /–
	Social Functioning	48.1 (26.1) / 53.9 (33.1)	74.4 (19.1) /–	47.3 (27.1) / 50.6 (26.9)	74.4 (19.1) /-
	Emotional Functioning	55.8 (29.3) / 67.3 (32.0)	67.6 (17.9) /–	43.0 (22.9) / 47.7 (25.6)	67.6 (17.9) /–
	Cognitive Functioning	69.2 (23.4) / 75.0 (25.8)	73.5 (18.6) /–	56.9 (23.3) / 59.9 (27.7)	73.5 (18.6) /–
	Communication	57.1 (27.2) / 67.3 (25.6)	81.9 (17.7) /–	48.1 (24.5) / 53.4 (26.9)	81.9 (17.7) /–
	Worry	42.7 (24.0) / **51.2 (23.9)**	78.1 (20.1) /–	35.6 (17.8) / **36.8 (21.0)**	78.1 (20.1) /–
	Daily Activities	**41.7 (30.1) /** 54.5 (33.6)	63.2 (22.5) /–	**31.6 (25.3) /** 38.1 (25.4)	63.2 (22.5) /–
	Family Relationships	**56.9 (22.8) /** 65.0 (25.6)	67.0 (19.4) /–	**59.0 (25.3) /** 62.5 (26.4)	67.0 (19.4) /–

PedsQL™-FIM	Summary Scores	Fathers (n = 13)	Reference Norms^2^	Mothers (n = 43/n = 42) ^6^	Reference Norms^2^
Past one month /past 7 days	FFSS	49.3 (24.6) / 59.7 (28.0)	67.6 (18.4) /–	45.3 (21.3) / 50.3 (22.4)	67.6 (18.4) /–
	*p*-value	**< 0.01**^**4**^	**< 0.05**^**5**^	**< 0.001**^**4**^	**< 0.001**^**5**^
	HRQoL-SS	57.7 (23.3) / 65.3 (27.3)	71.2 (14.9) /–	48.6 (19.8) / 52.4 (22.4)	71.2 (14.9) /–
	*p*-value	**< 0.05**^**4**^	> 0.05^5^	** < 0.005**^**4**^	**< 0.001**^**5**^
	Total Scale Score	53.6 (21.8) / 62.4 (25.1)	73.2 (13.6) /–	46.1 (18.3) / 50.0 (20.7)	73.2 (13.6) /–
	*p*-value	**< 0.01**^**4**^	**< 0.01**^**5**^	**< 0.005**^**4**^	**< 0.001**^**5**^

Data presented as mean (± SD). ^1^Reference norms are the U.S.A. community-based norms with a one-month recall period.^2^Reference norms are based on U.S.A. families with children without chronic conditions during a one-month recall period. ^4^*p*-value reflects the difference in summary scores between the past month and the past 7 days. ^5^*p*-value reflects the difference in parents’ one-month scores and the reference norm. ^6^n = 43 mothers over the past month and n = 42 mothers over the past 7 days. Notable items are marked in bold

### Siblings scored similarly to the reference on the PedsQL™-SC

Details are provided in Table 4 and Fig. 1. Siblings scored similarly to the reference norms over the past month, with the poorest scores in Emotional Functioning in the 18– < 25 age group, followed by the 13– < 18 and ≥ 25 age groups. Overall, the 18– < 25 age group had the poorest scores on all domains compared to other age groups over the past month and past 7 days. Siblings in the ≥ 25 age group scored significantly better than the reference norm on the Total Scale Score (*p* < 0.05) but not on the Psychosocial Health Score. There were no significant differences in summary scores between the reference norms and the other age groups.

**Table 4:** PedsQL™-SC Domain and Summary scores unaffected siblings versus norms: past month and past 7 days

PedsQL™-SC	Domain Scores	Siblings (13- < 18y) (n = 4)	Reference Norms^1^	Siblings (18- < 25y) (n = 5)	Reference Norms^1^	Siblings (≥ 25y) (n = 4)	Reference Norms^1^
Past one month/past 7 days	Physical Functioning	91.4 (9.0) / 93.0 (10.0)	90.7 (13.4) /–	**75.6 (25.4) / 78.8 (27.5)**	90.2 (12.5) /–	91.4 (6.4) / 94.5 (4.7)	88.7 (13.8) /–
	Social Functioning	97.5 (2.9) / 97.5 (2.9)	85.2 (17.0) /–	**76.0 (20.4) / 76.0 (20.4)**	88.4 (13.7) /–	98.8 (2.5) / 98.8 (2.5)	89.3 (12.8) /–
	Emotional Functioning	**78.8 (13.8) / 83.8 (14.4)**	80.4 (19.5) /–	**62.0 (25.9) / 62.0 (28.6)**	78.4 (17.7) /–	**80.0 (17.8) / 81.3 (11.1)**	78.7 (17.5) /–
	School/Work Functioning	91.2 (8.5) / 90.0 (10.8)	78.1 (17.8) /–	**63.0 (22.2) / 67.0 (19.9)**	84.0 (14.3) /–	95.0 (4.1) / 95.0 (4.1)	85.0 (14.6) /–

PedsQL™-SC	Summary Scores	Siblings(13- < 18y) (n = 4)	Reference Norms^1^	Siblings (18- < 25y) (n = 5)	Reference Norms^1^	Siblings (≥ 25y) (n = 4)	Reference Norms^1^
Past one month /past 7 days	Psychosocial Health Score	89.2 (6.2) / 90.4 (7.7)	81.2 (15.5) /–	67.0 (20.5) / 68.3 (21.5)	83.6 (12.7) /–	91.3 (7.4) / 91.7 (4.7)	84.3 (13.0) /–
	*p*-value	> 0.05^2^	> 0.05^3^	> 0.05^2^	> 0.05^3^	> 0.05^2^	>0.05^3^
	Total Scale Score	90.0 (10.8) / 91.1 (6.6)	84.5 (13.5) /–	67.0 (19.9) / 70.9 (22.7)	85.9 (11.2) /–	95.0 (4.1) / 92.4 (3.2)	85.9 (11.9) /–
	*p*-value	> 0.05^2^	> 0.05^3^	> 0.05^2^	> 0.05^3^	> 0.05^2^	**< 0.05**^**3**^

Data presented as Mean (± SD). ^1^ Reference norms are based on Dutch PedsQL-SC norms with a one-month recall period. ^2^*p*-value reflects the difference in summary scores between the past month and the past 7 days. ^3^*p*-value reflects the difference in sibling’ one-month scores and the reference norm. Notable items are marked in bold

### Differences in PedsQL™ scores based on recall periods

Fathers and mothers scored consistently poorer on all PedsQL™-FIM domains over the past month than over the past 7 days, with significant differences within their FFSS (*p* < 0.01 and *p* < 0.001, respectively), HRQoL-SS (*p* < 0.05 and *p* < 0.005, respectively), and Total Scale Scores (*p* < 0.01 and *p* < 0.005, respectively) (Table 3). The scores on all PedsQL™-SC domains of siblings were similar for the past month and past 7 days, without significant differences between their summary scores (Table 4).

### Identified main drivers from the impact questionnaires and semi-structured interviews

**Fig. 2:**
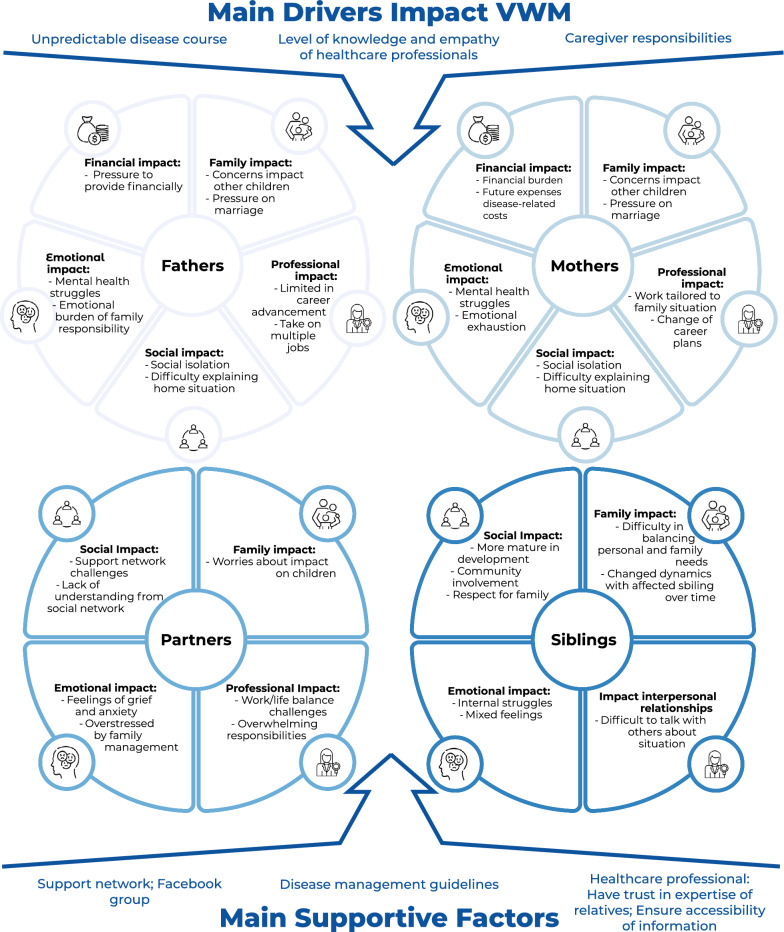
Conceptual overview: main drivers impact of VWM and supportive factors by unaffected family members

Table 5 provides details of the quantitative results. A total of 78 family members participated in the VWM impact questionnaire and semi-structured interviews. Saturation was reached with 58 coded transcripts. Three main drivers of VWM’s impact on unaffected family members were identified. Figure 2 presents an overview of the impact of the three main drivers and supportive factors on the different family members**.**

**Table 5:** Quantitative results of 5-point Likert scale of the impact inventory interview for parents

Question	N	Not at all % (n)	Not very % (n)	Neutral % (n)	Somewhat % (n)	Very % (n)	Median^3^	Mean (SD)
PARENTS								
Stress from first symptoms to diagnosis	78	1.3% (1)	7.7% (6)	2.6% (2)	14.1% (11)	**74.4% (58)**	4	3.5 (1.0)
Well-informed after diagnosis	77	20.8% (16)	32.5% (25)	6.5% (5)	16.9% (13)	23.4% (18)	1	1.9 (1.5)
Satisfied with diagnosis communication	78	12.8% (10)	11.5% (9)	19.5% (15)	30.8% (24)	25.6% (20)	3	2.5 (1.3)
Stress after diagnosis	78	0.0% (0)	1.3% (1)	3.8% (3)	11.5% (9)	**83.3% (65)**	4	3.8 (0.6)
Hope for finding a cure	78	10.3% (8)	7.7% (6)	7.7% (6)	28.2% (22)	46.1% (36)	3	2.9 (1.3)
Worried about acute deterioration	76	2.6% (2)	4.0% (3)	7.9% (6)	29.0% (22)	**56.6% (43)**	4	3.3 (1.0)
Frequency of worry about deterioration	78	1.3% (1)	6.4% (5)	15.4% (12)	32.1% (25)	**44.9% (35)**	3	3.1 (1.0)
Impact preventive measures on family life	77	1.3% (1)	5.2%(4)	9.1% (7)	36.4% (28)	**48.1% (37)**	3	3.3 (0.9)
Burden of caregiving^1^	78	19.2% (15)	6.4%(5)	23.1% (18)	34.6% (27)	16.7% (13)	3	2.2 (1.4)
Career satisfaction	77	9.1% (7)	7.9% (6)	24.7% (19)	27.3% (21)	31.2%(24)	3	2.6 (1.3)
Concerned about finances	78	11.5% (9)	29.5% (23)	12.8% (10)	33.3% (26)	12.8% (10)	2	2.1 (1.3)
Social acceptance of VWM	78	2.6% (2)	12.8% (10)	29.5% (23)	38.5% (30)	16.7% (13)	3	2.5 (1.0)
Current knowledge of VWM	77	0.0% (0)	5.2% (4)	3.9% (3)	40.3% (31)	50.7% (39)	4	3.4 (0.8)
Know more than healthcare professionals^2^	78	6.4% (5)	10.3% (8)	19.2% (15)	**30.8% (24)**	**33.3% (26)**	3	2.7 (1.2)
PARTNERS								
Stress from first symptoms to diagnosis	6	0.0% (0)	16.7% (1)	16.7% (1)	**67.7% (4)**	0.00% (0)	3	2.5 (0.8)
Well-informed after diagnosis	6	0.0% (0)	16.7% (1)	0.0% (0)	67.7% (4)	16.7% (1)	3	2.8 (1.0)
Satisfied with diagnosis communication	6	0.0% (0)	0.0% (0)	0.0% (0)	67.7% (4)	33.3% (2)	3	3.3 (0.5)
Stress after diagnosis	6	0.0% (0)	16.7% (1)	0.0% (1)	33.3% (2)	**50.0% (3)**	3.5	3.2 (1.2)
Hope for finding a cure	6	33.3 (2)	33.3 (2)	0.0% (0)	16.7 (1)	16.7 (1)	1	1.5 (1.6)
Worried about acute deterioration	6	0.0% (0)	16.7% (1)	0.0% (0)	**50.0% (3)**	33.3% (2)	3	3.0 (1.1)
Frequency of worry about deterioration	6	0.0% (0)	16.7% (1)	0.0% (0)	**67.7% (4)**	16.7% (1)	3	2.8 (1.0)
Impact preventive measures on family life	6	0.0% (0)	0.0% (0)	16.7% (1)	**50.0% (3)**	33.3% (2)	3	3.2 (0.8)
Burden of caregiving^1^	6	0.0% (0)	0.0% (0)	33.3% (2)	16.7% (1)	**50.0% (3)**	3.5	3.2 (1.0)
Career satisfaction	6	0.0% (0)	16.7% (1)	16.7% (1)	16.7% (1)	50.0% (3)	3.5	3.0 (1.3)
Concerned about finances	6	33.3% (2)	0.0% (0)	33.3% (2)	33.3% (2)	0.0% (0)	2	1.7 (1.4)
Social acceptance of VWM	6	0.0% (0)	**50.0 (3)**	16.7% (1)	0.00% (0)	33.3% (2)	1.5	2.2 (1.5)
Current knowledge of VWM	6	0.0% (0)	16.7% (1)	0.0% (0)	67.7% (4)	16.7% (2)	3	2.8 (1.0)
Know more than healthcare professionals^2^	6	0.0% (0)	0.0% (0)	33.3% (2)	**33.3% (2)**	**33.3% (2)**	3	3.0 (0.8)

Question	n	Not at all % (n)	Rarely % (n)	Neutral % (n)	Often % (n)	Almost always % (n)	Median^3^	Mean (SD)
SIBLINGS								
Thinks about sibling having VWM	13	0.0% (0)	0.0% (0)	23.1% (3)	**69.2% (9)**	7.7% (1)	3	2.9 (0.6)
Talks about it with friends	13	15.4% (2)	7.7% (1)	61.5% (8)	**15.4% (2)**	**0.0% (0)**	2	1.8 (0.9)
Talks about it with parents	13	15.4% (2)	15.4% (2)	38.5% (5)	**15.4% (2)**	**15.4% (2)**	2	2.0 (1.3)
Talks about it with someone else	13	23.1% (3)	15.4% (2)	46.2 (6)	**15.4% (2)**	**0.0% (0)**	2	1.5 (1.0)
Helps taking care of sibling	13	0.0% (0)	0.0% (0)	38.5% (5)	46.2% (6)	15.4% (2)	3	2.8 (0.7)
Misses school or work	13	53.9% (7)	38.5% (5)	7.7% (1)	0.0% (0)	0.0% (0)	0	0.5 (0.7)

^1^Scale: “absolutely not burdensome”, “not burdensome”, “neutral”, “burdensome”, “very burdensome”. ^2^Scale: “know absolutely less”, “know less”, “neutral”, “know more”, “know absolutely more”. ^3^Range of the median is for all questions 0–4. Notable items are marked in bold

N, total number; n, number

#### Driver 1: level of knowledge and empathy of healthcare professionals

A recurrent theme in the interviews was a sense amongst **parents and partners** that many healthcare professionals lack sufficient knowledge about VWM. Interviewees reported that they were given insufficient and often incorrect information immediately after the diagnosis, before being referred to a VWM specialist. They indicated that this led to great efforts to obtain information online, and that inaccurate and incomplete information added to their stress. Once well-informed, interviewees reported having to educate and inform physicians about VWM. Also, they frequently reported insensitivity and lack of empathy from healthcare professionals, especially in primary and secondary care settings. Interviewees highlighted that transparent, open, and empathic explanations are essential and mitigate emotional overload. They expressed a preference for information spread over multiple successive appointments. Interviewees considered it helpful if healthcare professionals refer to patient advocacy groups and the VWM Facebook group. Nearly all interviewees deemed peer support and information exchange necessary. Several also highlighted the need for crucial clinical information to be available in lay language.

**Parents** experienced a diagnostic delay with a mean of 2.7 (total range 0.13–13.9, SD = 3.7) years in 54% (39/71) of their children with VWM. According to parents, initial misdiagnosis occurred in 32% (24/75) of cases, often another type of leukodystrophy, neuromuscular disorder, or infectious disease. Parents reported high stress levels for the period between the onset of symptoms and the definitive diagnosis. Approximately 88% of parents (64/73) received their child’s diagnosis in the doctor’s office. Others were informed over the phone (10%, 7/72) or via email (3%, 2/72). Satisfaction with communication during diagnosis varied. Parents dissatisfied with communication at the time of the diagnosis had significantly lower PedsQL™-FIM scores: FFSS (*p* < 0.01), HRQoL-SS (*p* < 0.01), and Total Scale Scores (*p* < 0.005) than parents who were satisfied with the communication at the time of diagnosis. During the interviews, several parents emphasized the importance of being taken seriously and expressed frustration with being labeled as an ‘overprotective parent’. For example, a parent who was not satisfied with the communication of diagnosis commented: *“The neurologist was not interested in figuring out which leukodystrophy one it was and told us: They are all terminal, so, what is the point?”* Another parent who was satisfied with the communication mentioned that *“The neurologist has always been very helpful in terms of providing adequate information […]. She explains the situation, and is always open to questions, it is more of a dialogue.”* Around 53% (41/77) of the parents felt they lacked sufficient information on VWM immediately after receiving the diagnosis, although at the time of the survey, they reported a moderate improvement in their understanding of VWM. Parents obtained information on VWM from multiple resources. Approximately 59% (48/81) of parents obtained information through direct interactions with clinicians, nurses, or therapists, while 46% (37/80) obtained information from websites or literature, and 18% (14/80) relied on communication with other affected families. Additional sources, such as leukodystrophy newsletters and conferences, were reported by 9% (7/80) of parents. Around 64% (50/78) of parents felt more knowledgeable about VWM than standard healthcare professionals involved in their child’s care. During interviews, parents frequently expressed frustration with healthcare professionals. For example, a parent mentioned: *“It makes me feel uncomfortable that they do not know. Like the fact that I know more about this disease than they do. And they always will reply in like a strange way: they will be like “I have never heard of that.” They kind of look at me like I am making it up, like they say it in a way that they do not even think it [the disease] exists. I feel like it is very unprofessional […]. We have had ER doctors just dismiss it, basically, which bothers me a lot. But what do you do?”.*

50% (3/6) of the **partners** of adult VWM patients indicated difficulties to get diagnosed; one reported a diagnostic delay of 0.5 years, while two other partners experienced initial misdiagnoses. Approximately 83% of the partners (5/6) received their partner’s diagnosis in the doctor’s office, while one partner (17%, 1/ 6) received the diagnosis over email. All partners were somewhat to very satisfied with how the diagnosis was communicated. Several participants noted during the interviews that visiting a pediatric neurologist for a diagnosis can feel uncomfortable for adults. One partner, for example, noted that *“The strange thing was, that you end up in pediatric neurology, while my wife was already an adult, so naturally there was not much experience with patients like her.”* Most partners (67%, 4/6) obtained information directly from clinicians, nurses, or therapists and 33% (2/6) relied primarily on the internet. At the time of diagnosis and the survey, 83% of partners (5/6) felt somewhat to very well-informed. Around 66% (4/6) indicated that they have more knowledge of VWM than standard healthcare professionals. During the interviews, some partners expressed frustration that most information focuses on pediatric patients. One partner, for example, mentioned: *“We started to research about this disease and contacted groups with individuals who have it. But what annoys me is that they are almost entirely focused on children, so their experience is different from ours. It is completely different, and I do not have any information.”*

#### Driver 2: unpredictable disease course

Nearly **all family members** reported anxiety due to the unpredictable progression of the disease. The constant threat of acute episodes requires continuous vigilance for potential triggers, resulting in impaired daily functioning. Additionally, witnessing the progressive decline of their loved ones is frequently described as deeply distressing. Together, these factors contribute to chronic stress and emotional exhaustion. Supportive factors alleviating this impact mentioned by interviewees are clear guidelines for disease management including prevention strategies.

The threat of episodes of rapid decline makes that 86% (65/76) of the **parents** are somewhat to very worried, of which 77% (60/78) are often to almost always worried. To prevent these episodes of rapid deterioration, 96% (54/56) of the parents take preventive measures. Around 84% (65/77) mentioned some to very high impact of these preventive measures on family life. Parents indicated that these strategies often lead to social isolation, increased germaphobia, and reluctance to participate in social activities. As an example, one parent commented: *“The threat of an episode changed everything because I am in complete lockdown, so I cannot work from my office anymore. I do not visit the family anymore. Nobody comes to see us and when they are coming, we are only thinking about they could give [the VWM patient] an infection. So, this made me angry.*” Parents also highlighted the difficulties in balancing the restrictions imposed by preventive measures with their child’s desire for normal experiences and social interactions. They reported the difficulty in ensuring their child’s comfort and maintaining their abilities while also trying to encourage improvement, all without risking overexertion. One parent, for example, commented: *“It is mainly just really, really frustrating. It feels like you are doing everything you can to preserve function and that is the hard thing, it is like we are doing everything to preserve what [the VWM patient] has, not even improve it… It was just really frustrating and devastating that we were trying so hard and we were making such good progress in preserving function, and then an episode just wiped [the VWM patient] out and we are back to nothing.”*

83% (5/6) of **partners** are somewhat to very worried about episodes of rapid deterioration, with half (3/6) taking preventive measures to reduce the risk. 83% (5/6) report that these episodes have some to high impact on family life. Many of these partners (83%, 5/6) are frequently or almost always worried that such episodes could occur. One partner, for example, mentioned that “*After three days, the episode passed, and [the VWM patient] returned to [VWM patient’s] usual self. Now the question remains: will something similar happen again or not? This situation affects the children but also impacts* me.”

**Siblings** of affected VWM patients shared their anxiety about unintentionally triggering an episode and voiced concerns about their siblings’ future well-being. One sibling, for example, mentioned: “*I have known my whole life that an episode of acute decline can happen, I can prepare for it, and it is manageable, but I notice that when it happened, I was so emotional. I just kept crying and I thought, what is going on, but my parents did not really have the time to explain to me what was happening, because [the VWM patient] was being taken away by the ambulance at the time, but since, you know, there is nothing that can be done about it. You just have wait to see what will happen […]. Since then, I am always worried about if everything is going well”.*

#### Driver 3: the impact of caregiver responsibilities

A common theme among nearly **all family members** is the high demand for caregiving responsibilities. They reported challenges in finding reliable support due to long lists for home support services. Several interviewees expressed distrust toward external caregivers due to concerns about care quality. Supportive factors mentioned by interviewees include a good support network, both socially and financially. Additionally, engagement in community-based initiatives, such as participation in the Facebook group, was identified as a supportive factor.

The caregiving burden varies among **parents.** About 50% (40/78) indicated finding it (very) burdensome. Around 75% of the **parents** (47/63) highlighted that motor impairments have the most substantial effect on their daily living, followed by behavioral problems (11%, 7/63), seizures (6%, 4/63), tremors (6%, 4/63), communication difficulties (5%, 3/63), and headaches (5%, 3/63). Other issues mentioned by individual parents include sleep disturbances, premature menopause, cognitive impairments, (abdominal) pain, and incontinence (urinary and fecal). Additionally, 16% (10/63) expressed extra concerns about their child’s physical fragility, and 6% (4/63) found it particularly challenging that their child can no longer care for him- or herself. During the interviews, parents mentioned that motor impairments, in particular, impose a heavy physical and emotional impact, increasing their personal health risks and reducing personal time due to the demands of caregiving. For example, parents mentioned: *“The biggest challenges are primarily the physical symptoms, which affects both our child and us as parents. We have to take on most of the tasks for our child, which means that we are actually sacrificing our own health to support [VWM patient]. For example, this is just already by the fact that we often have to carry [VWM patient]”.*

Most **partners** (67%, 4/6) reported experiencing caregiving as (very) burdensome. The majority (67%, 4/6) reported behavioral problems as the most impactful on their daily living. Other issues mentioned by partners individually are migraines, loss of mobility, and premature menopause. During the interviews, partners mentioned diminished initiative and disinhibited behavior, which strain their relationships. One partner, for example, mentioned: “*You are constantly busy managing the household on your own, and when symptoms manifest as hurtful or aggressive behavior, it becomes especially challenging.”* Memory issues and paranoia further complicate caregiving. Another partner mentioned: *“The intellectual and emotional symptoms are the most difficult. Witnessing the changes in someone you care about, has a significant impact.”*

Around 45% (35/78) of the **parents** felt that VWM was not well understood and socially accepted by others**.** During the interviews, parents frequently reported experiencing social isolation as a result of the high demands associated with the disease. They found it challenging to explain the condition to friends and family, who want to help but often feel unsure about how to provide effective support. This leads to loss of friendships and weakened social connections. Supportive factors identified by parents include participation in Facebook groups that provide emotional support. For example, one parent mentioned: *“So I think talking to other families really helped me because they understand the situation. They have the same anger or sadness because their kids are going through it too.”* Help from their own parents was also a supportive factor, particularly when the affected child was still young.

50% of the **partners** (3/6) felt that VWM is not well understood and accepted socially. During the interviews, partners shared their frustration about repeatedly having to explain their home situation to friends and family, as the disease is often misunderstood. They reported challenges in building a support network, as friends often have their own commitments, and their parents are often elderly and unable to provide assistance. For example, a partner mentioned *“We have friends and neighbors who are willing to help, but they each have their own families and jobs. Our parents live far away and are dealing with health issues themselves, so we do not have anyone nearby who can come for a day to help us with our tasks.”*

Approximately 15% of the **siblings** (2/13) shared their experiences with friends, and 15% (2/14) shared these experiences with others. During the interviews, siblings expressed challenges in discussing their home situation as they try to avoid upsetting others. Discomfort with their home environment may contribute to fewer social interactions. One sibling, for example, mentioned”: “*I am too scared to ask anybody over because of [affected sibling] – or because our house is like really messy and I am like embarrassed, or maybe I do not even think about it. So, I will try… I am recently trying to get people to connect with me by like coming over, yeah, it is hard.”*

During the interviews**, parents** reported that their marriages are significantly impacted by the disease, as differences in coping and management strategies create tension. Moreover, the extensive demands of caregiving limit quality time between partners. Parents expressed concerns that unaffected siblings often take on caregiving roles, leading to unmet emotional needs. They worry that their unaffected child may receive less attention, become overly independent, or develop anxiety and/or frustration. One parent, for example, mentioned: *“I feel like [unaffected child] needs are not being met because we are focusing so much on [affected child]. She has a difficult time sharing with her friends because she already gives so much to her sibling, and then she ends up giving up everything.”* As a positive impact, parents also noted that their children had increased empathy and greater maturity than their peers.

**Partners** frequently expressed grief over their perceived loss of their loved one and also often worried about the impact on their children. For example, one partner mentioned: *“For me, the most important concern is how the children are affected by this, how they cope with it, and the potential negative consequences they might face.”* Partners frequently mentioned feeling overwhelmed by the responsibility of managing the family alone, with some expressing relief when their partner is admitted to a care facility.

Approximately 77% (10/13) of **siblings** reported frequently or almost always thinking about their affected sibling, and around 31% (4/13) indicated that they often to almost always share their feelings with their parents. During the interviews, they report complex emotions, including guilt about being healthy, not helping more, or not spending enough time with their affected sibling. Many feel a sense of loss and experience a changing relationship with their sibling when the disease progresses, especially in later stages of the disease. They tend to downplay their achievements and avoid self-pity. For example, a sibling mentioned: *“It is difficult for me to connect with [affected sibling] because I feel a lot of guilt.”* Some siblings reported that they cope with the situation by distancing themselves from the stressful home environment and sometimes engaging in maladaptive behaviors including drug abuse. Several siblings mentioned the strain of divided parental attention. One sibling, for example, mentioned: *“I try not to worry my parents about my feelings. If I am sad because I think about the future of my siblings, I try to keep it inside me and say: I cannot do anything, they cannot do anything.”* Despite these challenges, many siblings report greater independence, maturity, and empathy than their peers. They gain a broader perspective on life and develop a deeper respect for family relationships, often finding it essential to have support from other (un)affected siblings.

Around 58% (45/77) of the **parents** felt (very) satisfied with their current career. To meet caregiving demands, many parents modify their professional lives, often working from home to reduce infection risks and provide care. **Mothers** (median Likert scale score 2, IQR = 2–3) reported significantly poorer satisfaction with their professional development than **fathers** (median Likert scale score 3, IQR = 3–4) (*p* < 0.005). During the interviews, several mothers reported having to quit their jobs due to the overwhelming demands of caregiving, while many fathers described taking on multiple jobs to handle the increased financial burden, which they felt limited their opportunities for career advancement. One mother, for example, mentioned: *“I quit my job because I feel like I had to. I know a lot of people do not have that option. It is hard for us because my husband essentially has to have two jobs. He has to work extra so I can be with our child full-time.”* Around 46% (36/78) of the **parents** were from somewhat to very worried about finances. During the interviews, several parents mentioned financial burden and concerns about future expenses. The high costs of therapies, such as speech and physical therapy, add to this burden. Several parents also reported costly alternative treatments as a financial burden. **Mothers** (median Likert scale score 3, IQR = 1–3) had significantly higher scores on the level of financial worry than **fathers** (median Likert scale score 1, IQR = 1–3, *p* < 0.05). Several fathers indicated during the interviews that, while they did not experience financial strain due to stable, well-paying jobs with sufficient insurance coverage, they still felt pressure to provide financially. One father, for example, mentioned: *“I am the full-time provider, my wife does not work, so that is on me. I took responsibility for her to stay home, so yeah, every day, it weighs on my mind.”*

Most **partners** (67% 4/6) were somewhat to very satisfied with their career development. Around 33% (2/6) were somewhat concerned about their finances. During the interviews, several partners indicated difficulty achieving a work/life balance and had to stop working due to emotional exhaustion. One partner, for example, mentioned: *“Currently, I am too overwhelmed, and I have had to go on sick leave.”*

**Siblings,** who contribute to caregiving in some capacity, stated that this had little effect on their academic or work achievements, as 54% (7/13) never miss school or work.

## Discussion

This study addresses the impact of VWM on unaffected family members through standardized quality of life questionnaires, customized VWM impact inventories, and qualitative interviews. The impact of VWM varies across family members.

Regarding **parents**, mothers indicated a significantly poorer health-related quality of life, emotional well-being, and family functioning compared to the general population. They expressed dissatisfaction with their professional situation, often adapting their careers to meet their family needs. Fathers did not indicate a significantly poorer health-related quality of life and emotional well-being than the general population. In line with this, a study on the impact of MLD and Pontocerebellar Hypoplasia type 2 on unaffected relatives showed that mothers of these patients had significantly poorer health-related quality of life scores and were less content with their professional development than fathers (9). Fathers, however, had significantly poorer scores for family functioning. They expressed increased financial pressure and a heightened sense of responsibility to maintain family stability.

**Partners** reported emotional exhaustion and a significantly poorer quality of life and emotional well-being than the general population. They highlighted difficulties in managing family responsibilities, often feeling overwhelmed by the demands of the disease. Cognitive and behavioral symptoms of VWM patients further complicate these challenges. An explanation for the high burden on partners might be a suboptimal support network with elderly parents, and at the same time a demanding family situation with children and a partner with VWM. A study involving over 1,000 individuals with partners affected by chronic diseases indicated that the implications of the chronic disease impacted them beyond the caregiving burden [17]. This effect is especially pronounced when the illness is associated with social impairments. The impact of a severe neurological condition, such as VWM, on partners is largely overlooked, but appears to cause a significant burden.

Siblings did not indicate a significant impact of VWM on their quality of life on standardized questionnaires. Age may influence the varying impact of VWM on siblings, with the 13– < 18 and ≥ 25 groups scoring higher than the 18– < 25 group. The higher scores in the 13- < 18-year age group may reflect their greater difficulty discussing the disease, as confirmed in the qualitative interviews. Furthermore, measuring quality of life in children is more complex than in adults, as children often struggle with understanding abstract health and well-being concepts, especially in generic quality-of-life instruments [18]. Methods such as longitudinal interviews or peer-led discussions are needed to better capture and understand their perspectives. Interviews provided in-depth findings with many siblings describing challenges with social engagement, mixed emotions, guilt, and internal conflicts that made it difficult to share their feelings with others. Despite these challenges, siblings also reported positive influences on their empathy, self-development, and community involvement. This is in line with previous studies that reported mixed findings on the impact of chronic disease on siblings [19, 20].

Symptoms of VWM in patients are different for different ages of onset [5] and with that the impact for caregivers. For parents, 75% identified motor issues as the most impactful symptom for daily life. Notably, only half of the patients in the parent group experienced mobility problems, indicating that even mild motor issues can have a great impact for caregivers. By contrast, partners of adult patients identified behavioral challenges as the most impactful. These findings underline the importance of creating age-specific outcome measures for VWM in clinical trials, such as motor outcome measures for pediatric onset patients and behavioral outcome measures for adult onset patients.

Quantitative assessments of health-related quality of life in rare diseases often fail to fully capture patient and family perspectives by focusing solely on directly disease-related symptoms [21]. This was particularly the case for fathers and siblings, who scored higher than the general population in the Pain and Depression domain of the EQ5D-5L. Interviews revealed that they often felt unable to voice their struggles, believing their difficulties were less severe compared to the problems of their affected loved ones. This highlights the need for broader assessment approaches that consider the emotional and psychological burdens experienced by the entire family. Notably, the PedsQL™ is primarily designed for families with children, limiting its relevance for parents caring for adult patients, partners, and siblings, and may miss key aspects unique to their experience [13].

We found consistent differences in quality of life scores dependent on the recall period. The EQ5D assesses the present and the PedsQL™ covers the past month and past 7 days. PedsQL™ scores indicated better quality of life for the past 7 days than for the past month for parents but not for siblings. Short recall periods may be influenced more by diurnal or day-to-day fluctuation [22], while longer recall periods may either overestimate or underestimate a health state. Importantly, assessment of different aspects of quality of life may require varying recall periods [16]. For instance, assessment of psychological states often needs shorter recall periods, whereas measures of daily functioning or chronic pain may tolerate longer recall periods [16]. This issue is especially important for clinical trials, where selecting an inappropriate recall period can influence measures, potentially impacting the likelihood of detecting a treatment effect [23].

Qualitative interviews revealed deeper insight into the impact of VWM on unaffected family members and identified three main drivers: Perceived poor communication, acute episodes, and caregiver responsibilities. (1) Poor communication, lack of empathy, insufficient knowledge about VWM and limited guidance from healthcare professionals increase stress and uncertainty, worsened by misinformation online. (2) To avoid acute episodes of neurological decline strict preventive measures are necessary, which disrupt daily living and limit participation in normal activities. (3) Continuous caregiving responsibilities impair family functioning, impose financial burdens, hinder professional development, and often lead to social isolation. To address these drivers, healthcare professionals should express empathy and provide clear communication and straightforward clinical information. Offering symptom management resources in accessible language may help reduce uncertainty and alleviate anxiety. Referrals to advocacy groups and peer support networks can provide valuable emotional and informational support. Additionally, financial support programs are crucial for easing the financial burden on parents.

The impact of VWM on unaffected family members extends beyond a personal level, posing broader societal and economic challenges. Careers of parents and partners of VWM patients are often affected by the disease in the family primarily because of (1) time-consuming extra tasks and responsibilities, (2) infection-preventive measures, and (3) psychological and relationship changes. Health care costs for VWM patients are high and the loss of societal and economic productivity of unaffected family members adds to this. A study on leukodystrophies in the U.S.A. estimated that the costs of a leukodystrophy patient are 2 to 15 times higher than those of an average pediatric patient [24]. This substantial financial strain is driven by the severity of neurological impairments associated with the disease, such as the need for tube feeding and immobility due to VWM [25]. Moreover, episodes of rapid clinical decline frequently require inpatient hospitalization, which represents one of the most important drivers of healthcare resource use among leukodystrophy patients [26].

Limitations of our study, inherent to research on rare diseases, include a small sample size, incomplete or inconsistent responses from family members, and selection bias. In this study, Dutch or English-speaking participants were recruited from the existing VWM registry. This may have led to a skewed representation, as these families have the resources and knowledge to engage in research.

Importantly, appropriate reference values for the standardized quantitative questionnaires are not always available for all age groups, countries, and recall periods. In some instances, the results obtained could not be used for this reason (i.e. EQ5D-Y-3L).

Regional and cultural factors may affect the comparability of health-related quality of life outcomes across different populations. The EQ5D, for example, relies heavily on country-specific norms. In this study, we used the U.S.A. value set as reference data [13]. A sensitivity analysis comparing U.S.A. and Dutch value sets showed no significant differences between the two countries. This finding is consistent with EQ5D results from a multi-country study on MLD, showing that rare disease’s impact extends across international boundaries [11]. For the PedsQL™-FIM, we used reference data from a large U.S.A. cohort of families with healthy children [13]. Previous research on the impact of MLD and Pontocerebellar Hypoplasia type 2 suggests that variations in PedsQL™-FIM scores among parents of leukodystrophy patients across well-developed countries are minimal [9]. Due to the absence of age-appropriate U.S.A. reference data for the PedsQL™-SC, Dutch reference values were used. It is important to recognize that most studies focus primarily on patients in well-developed countries, and the experiences of families of leukodystrophy patients in developing countries may differ substantially.

Assessing the quality of life and drivers of VWM’s impact on unaffected family members is crucial for guiding patient management and therapy decisions, with the aim of enhancing the well-being of both patients and their families, and decreasing societal and economic loss. Including the entire family system of VWM patients provides a comprehensive and multi-faceted understanding of the disease’s impact and emphasizes the need for tailored approaches that address the full impact of a severe disease like VWM.

## Supplementary Information

Additional file 1.

## Data Availability

The datasets used and/or analyzed during the current study are available from the corresponding author on reasonable request.
