# Supplementary material for: The impact of vanishing white matter on unaffected family members
Source: Orphanet J Rare Dis. 2025 Aug 26;20:456. doi: 10.1186/s13023-025-03987-8 (PMC12379427; doi:10.1186/s13023-025-03987-8)
Supplement: Supplementary file 1 — Additional file 1. [file 13023_2025_3987_MOESM1_ESM.docx]

**Supplementary Material 1. Impact of disease inventory in Vanishing White Matter, semi-structured questionnaire for parents of patients**

**Impact of disease inventory in VWM: Questionnaire for Parents of patients**

**In case there are multiple children with VWM in your household, questions 1 - 36 should be answered for each affected child separately.**

**1. What was the age at onset of the first symptoms (in retrospect)**: ___ year(s) ___ month(s) ___ days
**2. To which medical doctors, hospitals, therapists etcetera was your child referred before the diagnosis was made?** Please provide a short chronological overview.

**3. Was there any diagnostic delay?**  ☐ Yes ☐ No ☐ I don’t know

**3.1 If yes, how long was the delay?** ___ year(s) ___ month(s)

**4. Was the patient initially misdiagnosed?** ☐ Yes ☐ No ☐ I don’t know

**4.1 If yes, please specify initial misdiagnosis**:

**5. How would you rate the stressfulness in the period from first symptoms to diagnosis?**

☐ Not stressful at all ☐ Not very stressful ☐ Neutral ☐ Somewhat stressful ☐ Very stressful  **6. How did you experience the period from first symptoms to diagnosis?** Give a short description.

**7. What was the age of your child at diagnosis of VWM?** ___ year(s) ___ month(s)

**8. How was the diagnosis communicated to you?**

☐ In-person conversation ☐ Via a letter ☐ Via e-mail ☐ Via telephone

**8.1** In case of an in-person conversation: **Where did the in-person conversation take place?**

☐ Doctor’s room ☐ Hospital ward/bed ☐ Hallway ☐ Elsewhere, namely

**9. In which hospital or center was the diagnosis made?**

**10. Who communicated the diagnosis to you?**

☐ Physician ☐ Other, namely

**11. How well-informed did you feel about VWM immediately after receiving the diagnosis?**

☐ Not informed at all ☐ Not very informed ☐ Neutral ☐ Somewhat informed ☐ Very well informed

**12. Were you satisfied with the way the diagnosis was communicated?**

☐ Not satisfied at all ☐ Not very satisfied ☐ Neutral ☐ Somewhat satisfied ☐ Very satisfied

**13. What did you find particularly helpful in the communication of the diagnosis?**

**14. What did you find particularly annoying in the communication of the diagnosis?**

**15. How stressful did you find the time immediately after the diagnosis?**

☐ Not stressful at all ☐ Not very stressful ☐ Neutral ☐ Somewhat stressful ☐ Very stressful

**16. How did you experience the period immediately after the diagnosis was made?** Give a short description.

**17. What is the impact of the fact that VWM cannot be cured?** Describe your experience.

**18. Do you feel that you have been given false hope by healthcare providers about the disease and its prognosis?**

**19. How would you rate your feeling/sense of hope for the discovery of a curative treatment?**

☐ Not hopeful at all ☐ Not very hopeful ☐ Neutral ☐ Somewhat hopeful ☐ Very hopeful

**20. The first therapeutic trial for VWM is currently ongoing.** **What are your feelings and thoughts about this?**

**21. Which symptom of your child’s disease has the highest impact on you as (a) parent(s)?**

**22. What is the impact of the decline in motor function?** Give a short description.

**23. What is the impact of the decline in cognitive capabilities?** Give a short description.

**24. What is the impact of changes in mood and/or behavior?** Give a short description.

**25. Did the patient experience any episodes of rapid deterioration?** ☐ Yes ☐ No ☐ I don’t know

If yes:

**25.1 Number of episodes with rapid deterioration**: _____

**25.2 When was the last episode of rapid deterioration?:**____ month _____ year
**26. How stressful was/were the episode(s) of rapid deterioration?**

☐ Not stressful at all ☐ Not very stressful ☐ Neutral ☐ Somewhat stressful ☐ Very stressful

**27. How worried are you about the threat of acute deterioration in everyday life?**

☐ Not worried at all ☐ Not very worried ☐ Neutral ☐ Somewhat worried ☐ Very worried

**28. How often are you worried about the threat of acute deterioration in everyday life?**

☐ Never ☐ Rarely ☐ Sometimes ☐ Often ☐ Almost always

**29. Please describe the impact of the threat of rapid deterioration on your daily (family) life.**

**30. How do the measures to prevent the acute episodes impact your daily (family) life?**

☐ No impact at all ☐ Not much impact ☐ Neutral ☐ Some impact ☐ High impact

**31. Please describe the impact of the preventive measures on your daily (family) life.**

**32. Please indicate the highest level of education your child achieved and select if special support was needed at school.**

☐ No school

☐ Kindergarten ☐ Without support ☐ With support

☐ Remedial/special kindergarten ☐ Without support ☐ With support

☐ Primary education ☐ Without support ☐ With support

☐ Primary special education ☐ Without support ☐ With support

☐ Secondary education/high school ☐ Without support ☐ With support

☐ Secondary special education ☐ Without support ☐ With support

**33. Who mainly takes care of the patient/your child?**

☐ Mother ☐ Father ☐ Both parents equally contribute ☐ Other, namely

**34. How much time per week do you spend on average with your child?**

____ hours/week

**35. Do you receive any support or home care for the daily care of your child?**

☐ No

**35.1 What is the reason for that?**

☐ Yes

**35.2 How many hours/week do you receive support?** _____ hours/wee

**35.3 By whom?** ☐ Home care ☐ Relative/family member ☐ Friend ☐ Other, namely

**36. How burdensome do you experience being a caregiver for your child?**

☐ Absolutely not burdensome ☐ Not burdensome ☐ Neutral ☐ Burdensome ☐ Very burdensome

**37. What is your highest achieved level of education:**

☐ Never been to school ☐ Primary education ☐ Primary special education ☐ Secondary education ☐ Secondary special education ☐ Tertiary education

**38. Current job**:

**39. How satisfied are you (mother) with your professional career?**

☐ Not satisfied at all ☐ Not very satisfied ☐ Neutral ☐ Somewhat satisfied ☐ Very satisfied
**40. Does the situation you have achieved professionally correspond to your originally planned goals?**

☐ Yes ☐ No ☐ I don’t know

If no:

**40.1 How did the medical situation of your child/the patient affected this?**

☐ Not at all ☐ A little bit ☐ Significant

**40.2 Can you shortly explain this?**

**41. Is your financial situation affected by the disease of your child?**

☐ Yes ☐ No ☐ I don’t know

**41.1** **Can you shortly explain this?**

**42. Are you worried about your financial situation?**

☐ Not worried at all ☐ Not very worried ☐ Neutral ☐ Somewhat worried ☐ Very worried

**43. Are you a single parent?**  ☐ Yes ☐ No ☐ I don’t know

**44. How many biological children do you have?**

44.1 Number of biological children affected with VWM:

44.2 Number of biological children who are deceased:

44.2.1. What was the cause of death?

**45. How many children belong to your family who are not biologically yours?**

45.1 Number of non-biological children affected with VWM:

45.2 Number of non-biological children who are deceased:

44.2.1. What was the cause of death?

**46. How many children are currently living in your household?**

46.1 Number of children affected with VWM:

**47. Are there any other people part of your household besides you, your partner, and the above mentioned children?**

☐ Yes, namely ☐ No

**48. In the case of healthy sibling(s), what is the positive and/or negative impact of the disease on the healthy sibling(s)?** Choose which item(s) apply.

☐ Shows behavioral problems, such as depression, aggression, or hyperactivity

☐ His/her needs are not met due to the demanding care of the affected sibling

☐ Helps a lot in daily care of the affected sibling

☐ Takes a lot of family responsibilities, in other areas too

☐ Other, namely

**49. Who are the most important persons within your support network?** Select max 3.

☐ Partner ☐ Family ☐ Friends ☐ Neighbors ☐ Other affected family/families ☐ Support group ☐ Patient organizations ☐ Facebook groups ☐ Religion ☐ Other, namely

**50. How do you rate the social acceptance of having a child with VWM?**

☐ Not good at all ☐ Not very good ☐ Neutral ☐ Good ☐ Very good

**51. Which reactions do you encounter most frequently in everyday life with your affected child (please put a maximum of three crosses)?**

☐ Sympathy ☐ Pity ☐ Incomprehension/misunderstanding ☐ Contempt ☐ Other, namely

**52. How well-informed on VWM are you at the moment?**

☐ Not informed at all ☐ Not very informed ☐ Neutral ☐ Well-informed ☐ Very well-informed

**53. Where did you gained most of your knowledge?**

☐ Conversations with clinicians/therapists/nurses ☐ Conversations with other affected families ☐ Via websites ☐ Via professional literature ☐ Other, namely

**54. In your opinion, what is needed to become even better informed about VWM?**

**55. From experience we know that parents become experts on VWM after the diagnosis is made.** **In general, do you know more or less about VWM compared to the healthcare professionals involved in the care of your child?**

☐ I absolutely know more than most of the healthcare professionals

☐ I know more than most of the healthcare professionals

☐ Neutral

☐ I know less than most of the healthcare professionals

☐ I absolutely know less than most of the healthcare professionals

**56. What is your experience with the level of knowledge/expertise on VWM of healthcare professionals involved in the care of your child?** Give a short description.

**Supplementary Material 2. Impact of disease inventory in Vanishing White Matter, semi-structured questionnaire for partners of adult patients**

**Impact of disease inventory in VWM: Questionnaire for partners of adult patients**

**1. What was the age at onset of the first symptoms (in retrospect)**: ___ year(s) ___ month(s) ___ days

**2. To which medical doctors, hospitals, therapists etcetera was your partner referred before the diagnosis was made?** Please provide a short chronological overview.

**3. Was there any diagnostic delay?**  ☐ Yes ☐ No ☐ I don’t know

**3.1 If yes, how long was the delay?** ___ year(s) ___ month(s)

**4. Was the patient initially misdiagnosed?** ☐ Yes ☐ No ☐ I don’t know

**4. 1 Please specify initial misdiagnosis**:

**5. Did your relationship start before the first clear symptoms of VWM?**

☐ Yes ☐ No

If no:

**5.1. Did you relationship start before the diagnosis of VWM?**

☐ Yes ☐ No

**6. How would you rate the stressfulness in the period from first symptoms to diagnosis?**

☐ Not stressful at all ☐ Not very stressful ☐ Neutral ☐ Somewhat stressful ☐ Very stressful

**7. How did you experience the period from first symptoms to diagnosis?** Give a short description.

**8. What was the age of your partner at diagnosis of VWM?** ___ year(s) ___ month(s)

**9. How was the diagnosis communicated to you?**

☐ In-person conversation ☐ Via a letter ☐ Via e-mail ☐ Via telephone

**9.1 In case of an in-person conversation, where did the in-person conversation take place?**

☐ Doctor’s room ☐ Hospital ward/bed ☐ Hallway ☐ Elsewhere, namely

**10. In which hospital or center was the diagnosis made?**

**11. Who communicated the diagnosis to you?**

☐ Physician ☐ Other, namely

**12. How well-informed did you feel about VWM immediately after receiving the diagnosis?**

☐ Not informed at all ☐ Not very informed ☐ Neutral ☐ Somewhat informed ☐ Very well informed

**13. Were you satisfied with the way the diagnosis was communicated?**

☐ Not satisfied at all ☐ Not very satisfied ☐ Neutral ☐ Somewhat satisfied ☐ Very satisfied

**14. What did you find particularly helpful in the communication of the diagnosis?**

**15. What did you find particularly annoying in the communication of the diagnosis?**

**16. How stressful did you find the time immediately after the diagnosis?**

☐ Not stressful at all ☐ Not very stressful ☐ Neutral ☐ Somewhat stressful ☐ Very stressful

**17. How did you experience the period immediately after the diagnosis was made?** Give a short description.

**18. What is the impact of the fact that VWM cannot be cured?** Describe your experience.

**19. Do you feel that you have been given false hope by healthcare providers about the disease and its prognosis?**

**20. How would you rate your feeling/sense of hope for the discovery of a curative treatment?**

☐ Not hopeful at all ☐ Not very hopeful ☐ Neutral ☐ Somewhat hopeful ☐ Very hopeful

**21. The first therapeutic trial for VWM is currently ongoing.** **What are your feelings and thoughts about this?**

**22. Which symptom of your partner’s disease has the highest impact on you?**

**23. What is the impact of the decline in motor function?** Give a short description.

**24. What is the impact of the decline in cognitive capabilities?** Give a short description.

**25. What is the impact of changes in mood and/or behavior?** Give a short description.

**26. Did the patient experience any episodes of rapid deterioration?** ☐ Yes ☐ No ☐ I don’t know

If yes:

**26.1 Number of episodes with rapid deterioration**: _____

**26.2 When was the last episode of rapid deterioration?:** _____ month _____ year
**27. How stressful was/were the episode(s) of rapid deterioration?**

☐ Not stressful at all ☐ Not very stressful ☐ Neutral ☐ Somewhat stressful ☐ Very stressful

**28. How worried are you about the threat of acute deterioration in everyday life?**

☐ Not worried at all ☐ Not very worried ☐ Neutral ☐ Somewhat worried ☐ Very worried

**29. How often are you worried about the threat of acute deterioration in everyday life?**

☐ Never ☐ Rarely ☐ Sometimes ☐ Often ☐ Almost always

**30. Please describe the impact of the threat of rapid deterioration on your daily (family) life.**

**31. How do the measures to prevent the acute episodes impact your daily (family) life?**

☐ No impact at all ☐ Not much impact ☐ Neutral ☐ Some impact ☐ High impact

**32. Please describe the impact of the preventive measures on your daily (family) life.**

**33. How much time per week do you spend on average with your partner?**

____ hours/week

**34. Do you receive any support or home care for the daily care of your partner?**

☐ No

**34.1 What is the reason for that?**

☐ Yes

**34.2 How many hours/week do you receive support?** _____ hours/week

**34.3 By whom?** ☐ Home care ☐ Relative/family member ☐ Friend ☐ Other, namely

**35. How burdensome do you experience being a caregiver for your partner?**

☐ Absolutely not burdensome ☐ Not burdensome ☐ Neutral ☐ Burdensome ☐ Very burdensome

**36. What is your highest achieved level of education:**

☐ Never been to school ☐ Primary education ☐ Primary special education ☐ Secondary education ☐ Secondary special education ☐ Tertiary education

**37. Current job:**

**38. How satisfied are you with your professional career?**

☐ Not satisfied at all ☐ Not very satisfied ☐ Neutral ☐ Somewhat satisfied ☐ Very satisfied
**39. Does the situation you have achieved professionally correspond to your originally planned goals?**

☐ Yes ☐ No ☐ I don’t know

If no:

**39.1 How did the medical situation of your partner affected this?**

☐ Not at all ☐ A little bit ☐ Significant

**39.2 Can you shortly explain this?**

**40. Is your financial situation affected by the disease of your partner?**

☐ Yes ☐ No ☐ I don’t know

**40.1** **Can you shortly explain this?**

**41. Are you worried about your financial situation?**

☐ Not worried at all ☐ Not very worried ☐ Neutral ☐ Somewhat worried ☐ Very worried

**42. Do you have children?** ☐ Yes ☐ No

**43. Number of children living in your household:** ________

**44. Are there any other people part of your household besides you, your partner, and the above mentioned children?**

☐ Yes, namely ☐ No

**45. What is the positive and/or negative impact of the disease on the children?** Choose which item(s) apply.

☐ Shows behavioral problems, such as depression, aggression, or hyperactivity

☐ His/her needs are not met due to the demanding care of your partner

☐ Helps a lot in daily care of your partner

☐ Takes a lot of family responsibilities, in other areas too

☐ Other, namely

**46. Who are the most important persons within your support network?** Select max 3.

☐ Family ☐ Friends ☐ Neighbors ☐ Other affected family/families ☐ Support group ☐ Patient organizations ☐ Facebook groups ☐ Religion ☐ Other, namely

**47. How do you rate the social acceptance of having a partner with VWM?**

☐ Not good at all ☐ Not very good ☐ Neutral ☐ Good ☐ Very good

**48. Which reactions do you encounter most frequently in everyday life with your partner (please put a maximum of three crosses)?**

☐ Sympathy ☐ Pity ☐ Incomprehension/misunderstanding ☐ Contempt ☐ Other, namely

**49. How well-informed on VWM are you at the moment?**

☐ Not informed at all ☐ Not very informed ☐ Neutral ☐ Well-informed ☐ Very well-informed

**50. Where did you gained most of your knowledge?**

☐ Conversations with clinicians/therapists/nurses ☐ Conversations with other affected families ☐ Via websites ☐ Via professional literature ☐ Other, namely

**51. In your opinion, what is needed to become even better informed about VWM?**

**52. From experience we know that family become experts on VWM after the diagnosis is made. In general, do you know more or less about VWM compared to the healthcare professionals involved in the care of your partner?**

☐ I absolutely know more than most of the healthcare professionals

☐ I know more than most of the healthcare professionals

☐ Neutral

☐ I know less than most of the healthcare professionals

☐ I absolutely know less than most of the healthcare professionals

**53. What is your experience with the level of knowledge/expertise on VWM of healthcare professionals involved in the care of your partner?** Give a short description.

**Supplementary material 3. Impact of disease inventory in Vanishing White Matter, semi-structured questionnaire for unaffected siblings of patients**

**Supplementary Material 3. Impact of disease inventory in Vanishing White Matter, semi-structured questionnaire for unaffected siblings of patients**

**Impact of disease inventory in VWM: Questionnaire for siblings**

**1. Do you think about your brother/sister having Vanishing White Matter?**

☐ Never ☐ Rarely ☐ Sometimes ☐ Often ☐ Almost always

**1.1 Can you explain?**

**2. Can you describe what you are thinking about?**

**3. How would you describe your relationship with your brother/sister?**

**4. Do you tell your friends about the condition of your brother/sister?**

☐ Never ☐ Rarely ☐ Sometimes ☐ Often ☐ Almost always

**4.1 Why is that?**

**5. Do you talk with your parents about the condition of your brother/sister?**

☐ Never ☐ Rarely ☐ Sometimes ☐ Often ☐ Almost always

**5.1 Can you explain?**

**6. Or with someone else?**

☐ Never ☐ Rarely ☐ Sometimes ☐ Often ☐ Almost always

**6.1 Who is that or who are they?**

**7. Do you help to take care of your brother/sister?**

☐ Never ☐ Rarely ☐ Sometimes ☐ Often ☐ Almost always

**7.1 Why is that?**

**8. Can you describe what you are doing to help?**

**9. How long does that take?**

**10. How are you doing in school or at work?**

☐ Poorly ☐ Average ☐ Very well

**10.1 Can you explain why?**

**11. Do you participate in activities outside school or work?**

☐ No ☐ yes

**11.1 If yes, how much time per week?**

**12. Is that more or less than your peers?**

☐ More ☐ The same ☐ Less

**12.1 Can you explain why?**

**13. Do you sometimes miss school or work?**

☐ Never ☐ Rarely ☐ Sometimes ☐ Often ☐ Almost always

**13.1 Can you mention the most common reasons for missing school?**

**14. Does having a brother/sister with Vanishing White Matter have a negative impact on your life in any way?**

☐ Yes ☐ No ☐ I don’t know

**14.1 Can you explain?**

**15. Is there a positive side to having a brother/sister with Vanishing White Matter?**

☐ Yes ☐ No ☐ I don’t know

**15.1 Can you explain?**

**16. If there is anything else you would like to share?**

**Supplementary Tables**

**Supplementary Table 1. EQ5D-5L/Y-3L dimension score per level for unaffected family members and reference norms**

| **Dimension** | **Reference Norm¹** | **Fathers (n=29)** | **Mothers**  **(n=52)** | **Partners**  **(n=6)** | **Unaffected siblings ≥18 y**  **(n=9)** | **Unaffected siblings <18 y**  **(n=4) ²** |
| --- | --- | --- | --- | --- | --- | --- |
| **Mobility** |  |  |  |  |  |  |
| **MO1(%)** | 70.6 | 82.8 | 76.9 | 66.7 | 88.9 | 100.0 |
| **MO2 (%)** | 17.9 | 10.4 | 7.7 | 16.7 | 11.1 | 0.0 |
| **MO3 (%)** | 8.5 | 0.00 | 3.9 | 0.0 | 0.0 | 0.0 |
| **MO4 (%)** | 2.1 | 3.5 | 3.9 | 16.7 | 0.0 | NA |
| **MO5 (%)** | 0.9 | 3.5 | 7.7 | 0.0 | 0.0 | NA |
| **Self-Care** |  |  |  |  |  |  |
| **SC1 (%)** | 87.0 | 86.2 | 82.7 | 83.3 | 88.9 | 100.0 |
| **SC2 (%)** | 8.6 | 10.4 | 5.8 | 16.7 | 11.1 | 0.0 |
| **SC3 (%)** | 3.2 | 0.0 | 5.8 | 0.0 | 0.0 | 0.0 |
| **SC4 (%)** | 0.7 | 0.0 | 0.00 | 0.0 | 0.0 | NA |
| **SC5 (%)** | 0.5 | 3.5 | 5.8 | 0.0 | 0.0 | NA |
| **Usual Activities** |  |  |  |  |  |  |
| **UA1 (%)** | 68.8 | 75.9 | 61.5 | 50.0 | 66.7 | 100.0 |
| **UA2 (%)** | 19.1 | 13.8 | 13.5 | 16.7 | 33.3 | 0.0 |
| **UA3 (%)** | 9.3 | 6.9 | 11.5 | 16.7 | 0.0 | 0.0 |
| **UA4 (%)** | 2.3 | 3.5 | 9.6 | 0.0 | 0.0 | NA |
| **UA5 (%)** | 0.5 | 0.00 | 3.9 | 16.7 | 0.0 | NA |
| **Pain/Discomfort** |  |  |  |  |  |  |
| **PD1 (%)** | 37.1 | 62.1 | 44.2 | 33.3 | 77.8 | 100.0 |
| **PD2 (%)** | 37.7 | 20.7 | 28.9 | 33.3 | 11.1 | 0.0 |
| **PD3 (%)** | 19.0 | 13.8 | 17.3 | 33.3 | 11.1 | 0.0 |
| **PD4 (%)** | 5.2 | 3.5 | 7.7 | 0.0 | 0.0 | NA |
| **PD5 (%)** | 1.1 | 0.00 | 1.9 | 0.0 | 0.0 | NA |
| **Depression/ Anxiety** |  |  |  |  |  |  |
| **AD1 (%)** | 48.9 | 55.2 | 26.9 | 0.0 | 55.6 | 50.0 |
| **AD2 (%)** | 26.0 | 27.6 | 26.9 | 33.3 | 22.2 | 50.0 |
| **AD3 (%)** | 17.3 | 10.3 | 28.9 | 66.7 | 22.2 | 0.0 |
| **AD4 (%)** | 5.0 | 3.5 | 11.5 | 0.0 | 0.0 | NA |
| **AD5 (%)** | 2.9 | 3.5 | 5.8 | 0.0 | 0.0 | NA |

N.B. Due to rounding, percentage may not add up to 100%. ¹ Reference norms are based on the U.S.A. EQ5D-5L scores. **²**Scores of the EQ5D-Y-3L ; y, years; NA, not applicable

**Supplementary Table 2. Sensitivity analysis of EQ-5D Utility Index scores: comparison of U.S.A. and The Netherlands value sets among unaffected family members**

| **Country** | **Utility Index** | **Fathers**  **(n=29)** | **Mothers**  **(n=52)** | **Partners**  **(n=6)** | **Unaffected siblings ≥18 y (n=9)** |
| --- | --- | --- | --- | --- | --- |
| **U.S.A. (0.883)** | Median (IQR) | 0.94 (0.82-1.00) | 0.82 (0.53-0.94) | 0.73 (0.64-0.83) | 0.94 (0.88-1.00) |
|  | Mean (±SD) | 0.84 (0.28) | 0.70 (0.32) | 0.71 (0.17) | 0.90 (0.15) |
|  | 95% CI | 0.74-0.95 | 0.61-0.79 | 0.53-0.88 | 0.79-1.00 |
|  | P-value | >0.05 | <0.001 | <0.05 | >0.05 |
| **NL (~0.869)** | Median (IQR) | 0.88 (0.78-1.00) | 0.77 (0.53-0.88) | 0.71 (0.62-79) | 0.89 (0.84-1.00) |
|  | Mean (±SD) | 0.83 (0.26) | 0.68 (0.29) | 0.69 (0.13) | 0.88 (0.13) |
|  | 95% CI | 0.73-0.93 | 0.60-0.76 | 0.56-0.82 | 0.79-0.98 |
|  | P-value | >0.05 | <0.001 | <0.05 | >0.05 |

~ Estimation similar to the mean reported by Versteegh and colleagues (16), y, years
